# Lignocellulose conversion for biofuel: a new pretreatment greatly improves downstream biocatalytic hydrolysis of various lignocellulosic materials

**DOI:** 10.1186/s13068-015-0419-4

**Published:** 2015-12-24

**Authors:** Seung Gon Wi, Eun Jin Cho, Dae-Seok Lee, Soo Jung Lee, Young Ju Lee, Hyeun-Jong Bae

**Affiliations:** Bio-Energy Research Center, Chonnam National University, Gwangju, 500-757 Republic of Korea; Gwangju Center, Korea Basic Science Institute, Gwangju, 500-757 Republic of Korea; Department of Bioenergy Science and Technology, Chonnam National University, Gwangju, 500-757 Republic of Korea

**Keywords:** Bioethanol, Lignocellulosic biomass, Pretreatment, Energy efficiency

## Abstract

**Background:**

Lignocellulosic biomass is an attractive renewable resource for future liquid transport fuel. Efficient and cost-effective production of bioethanol from lignocellulosic biomass depends on the development of a suitable pretreatment system. The aim of this study is to investigate a new pretreatment method that is highly efficient and effective for downstream biocatalytic hydrolysis of various lignocellulosic biomass materials, which can accelerate bioethanol commercialization.

**Results:**

The optimal conditions for the hydrogen peroxide–acetic acid (HPAC) pretreatment were 80 °C, 2 h, and an equal volume mixture of H_2_O_2_ and CH_3_COOH. Compared to organo-solvent pretreatment under the same conditions, the HPAC pretreatment was more effective at increasing enzymatic digestibility. After HPAC treatment, the composition of the recovered solid was 74.0 % cellulose, 20.0 % hemicelluloses, and 0.9 % lignin. Notably, 97.2 % of the lignin was removed with HPAC pretreatment. Fermentation of the hydrolyzates by *S. cerevisiae* resulted in 412 mL ethanol kg^−1^ of biomass after 24 h, which was equivalent to 85.0 % of the maximum theoretical yield (based on the amount of glucose in the raw material).

**Conclusion:**

The newly developed HPAC pretreatment was highly effective for removing lignin from lignocellulosic cell walls, resulting in enhanced enzymatic accessibility of the substrate and more efficient cellulose hydrolysis. This pretreatment produced less amounts of fermentative inhibitory compounds. In addition, HPAC pretreatment enables year-round operations, maximizing utilization of lignocellulosic biomass from various plant sources.

**Electronic supplementary material:**

The online version of this article (doi:10.1186/s13068-015-0419-4) contains supplementary material, which is available to authorized users.

## Background

The use of lignocellulosic bioenergy can reduce fossil fuel dependence and greenhouse gas emissions [1–3]. The largest proportion of petroleum consumption is for transportation, and bioenergy is clearly the only sustainable, low-cost, large-scale fuel production option [4–7]. However, producing bioethanol from lignocelluloses has limitations, given the inherent inefficiency of extracting lignin, a highly recalcitrant lignocellulosic cell wall polymer, under mild conditions and with minimal loss of polysaccharides [8, 9]. The success of lignocellulosic bioethanol will depend on the development of simple pretreatment technologies that effectively delignify a diverse portfolio of lignocellulosic biomass feedstocks. Reducing the enzyme cost while enhancing cellulose hydrolysis efficiency is another important consideration when developing suitable pretreatment technologies [10–12].

The enzymatic hydrolysis of lignocellulosic biomass is influenced by several factors, including lignin and hemicelluloses contents, cellulose crystallinity, degree of polymerization, accessible surface area, and pore volume. However, lignin has been believed to be a major hindrance in enzymatic hydrolysis [13, 14]. Because lignin forms a physical barrier to restrict the acess of cellulases to the cellulose. Delignification increases cell wall porosity, rendering the biomass more amenable to enzymatic hydrolysis. Selective lignin removal can minimize cellulose degradation and thus enhance enzymatic hydrolysis [15–17].

Maximizing the utilization of lignocellulosic biomass materials from various plant sources through year-round operation is another important requirement for developing effective pretreatment technologies. Many herbaceous biomass crops become available only for a specific time in the year. In addition, even though lignocellulosic biomass can be supplied annually, most pretreatments have been optimized for a specific model biomass, which has challenged the effective use of other biomass sources [18–21]. As cell walls in biomass feedstocks differ in structure and chemical composition, one pretreatment method will not necessarily fit all applications. Therefore, developing a pretreatment technology that is effective over a wide range of biomass materials is important.

A variety of pretreatment methods have been applied for pretreating lignocellulosic biomass, but only a few of them seem to be promising. These pretreatment methods include steam explosion, ammonia fiber expansion (AFEX), dilute acid, and ionic-liquid pretreatment. Various pretreatment processes for lignocellulosic biomass, and their advantages and disadvantages are summarized in Table 1 [22–25]. An ideal pretreatment should be cheap, as much as removal lignin, effective for various lignocellulosic substrates, minimal glucan loss, and less inhibitor generation. Consequently, there is currently no single pretreatment technology that is potentially acceptable for the multiple biomass conversion.

**Table 1:** Summary of pretreatment methods

Pretreatment methods	Conditions	Advantage	Disadvantage	Reference
Steam explosion	1.3 Mpa 190 °C 15 min	Hemicelluloses removal	Acid catalyst needed Formation of inhibitors	[22]
AFEX	Anhydrous ammonia 60–100 °C 250–300 psi	Lignin removal Cellulose decrystallization	High energy	[23]
Dilute acids	HCl/H_2_SO_4_ 130–200 °C	Hemicelluloses removal	Formation of inhibitors Neutralization step	[24]
Ionic-liquid	90–200 °C 1–24 h	Break-up cellulose crystalline	Expensive	[25]
HPAC	CH_3_COOH:H_2_O_2_ 80 °C, <2 h	Effectively lignin removal	Hemicellulose loss	Present work

In this study, we introduce a new pretreatment method that is more efficient and effective for downstream biocatalytic hydrolysis of various lignocellulosic materials, which will accelerate bioethanol commercialization. This developed hydrogen peroxide (H_2_O_2_)-acetic acid (CH_3_COOH) (HPAC) pretreatment highly removes lignin without the use of high temperatures or strong acids. It can be applied to multiple lignocellulosic materials, reduces enzyme loading and downstream enzymatic hydrolysis time, and lowers generation of fermentation inhibitors during the process. We propose that the HPAC is a highly effective pretreatment to bioconvert lignocellulosic biomass. We provide data from our work on lignin removal, enzymatic hydrolysis, and fermentation of various lignocellulosic biomass materials.

## Results and discussion

### Features of the HPAC pretreatment

**Fig. 1:**
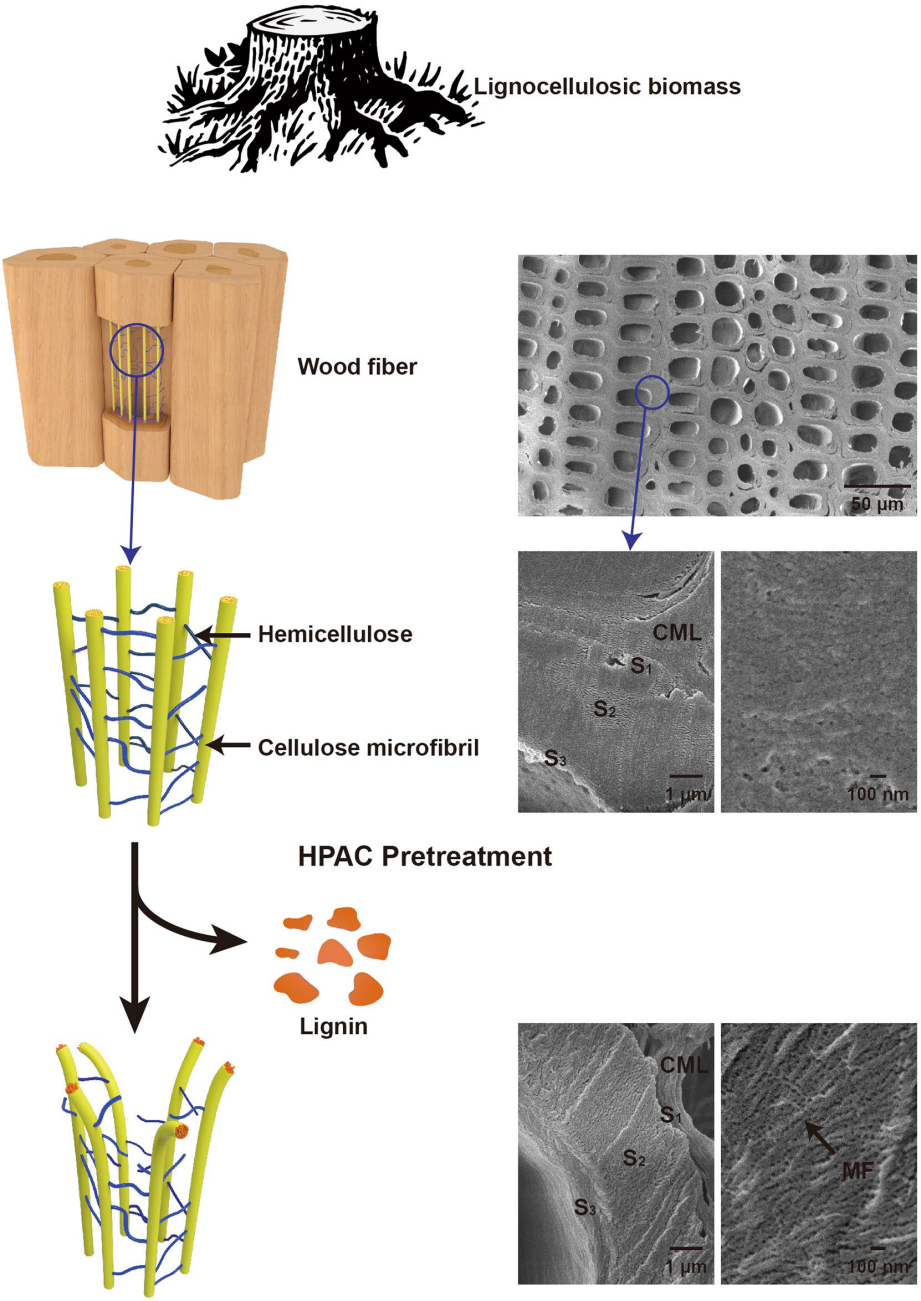
A proposed conceptual model for the mechanism of enhanced enzymatic saccharification of lignocellulosic biomass by delignification using the hydrogen peroxide (H_2_O_2_)-acetic acid (CH_3_COOH) (HPAC) pretreatment

Our integrated approach to the new HPAC pretreatment method is explained in Fig. 1. It is a simple procedure involving mixing H_2_O_2_ and CH_3_COOH to form a reagent that effectively removes lignin from lignocellulosic biomass through partial hydrolysis of lignin bonds. Pretreatment efficiency was evaluated based on observations of lignin removal, enzymatic hydrolysis, and fermentation of rice straw, pine wood, and oak wood.

### Optimal conditions

**Fig. 2:**
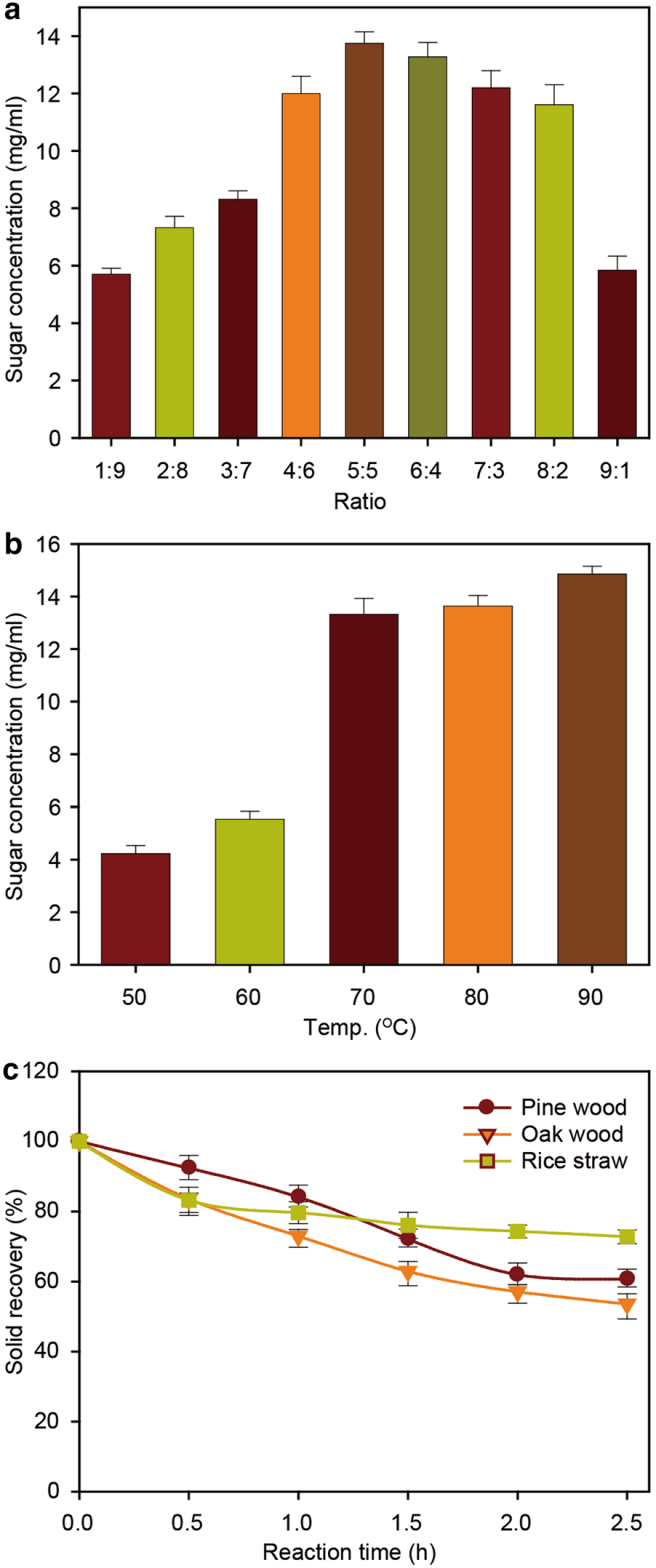
**a** Effect of different volume ratios of hydrogen peroxide/acetic acid. (*Conditions:* temperature, 80 °C; time, 2 h) **b** Effects of temperature on relative conversion to sugars. (*Substrate*: pine wood, *Conditions*: volume ratio of hydrogen peroxide/acetic acid, 5:5; time, 2 h) **c** Solid recovery after the HPAC pretreatment

To optimize the pretreatment conditions, we first measured the ratio of H_2_O_2_ to CH_3_COOH. The selected volume ratios were 1:9, 2:8, 3:7, 4:6, 5:5, 6:4, 7:3, 8:2, and 9:1. An equal volume mixture (5:5) was most effective (Fig. 2a and Additional file 1: Figure S1).

Other important factors are reaction temperature and time [26]. To verify the importance of these factors, we investigated the optimum pretreatment conditions necessary for enhancing enzymatic hydrolysis. Experiments were done to investigate their effects on pretreatment by fixing the volume ratio of H_2_O_2_ and CH_3_COOH at 5:5. The effects of temperature and time on cellulose content are shown in Fig. 2b, c. Optimal pretreatment conditions for all three biomass materials were a temperature of 80 °C and a treatment time of 2 h, which gave a high yield of total sugars (Fig. 2b and Additional file 2: Figure S2). Generally, a longer pretreatment time and higher temperature increase delignification efficiency; however, these conditions use more energy, which is also an important factor to consider [27]. The acid pretreatment is usually carried out at high temperature, which causes metal corrosion, and this is also an important consideration when designing pretreatment reactors [28].

We also investigated total solid recovery after pretreatment. As expected, the recovery of solids decreased with increasing pretreatment time (Fig. 2c). Total recovery of solids after 2 h pretreatment was 58.8–75.2 %, based on the initial biomass weight. The decrease in water-insoluble solids (WIS) was mainly due to solubilization of hemicelluloses and lignin-derived compounds. Therefore, the optimal conditions for the HPAC pretreatment were 80 °C, 2 h, and an equal volume mixture of H_2_O_2_ and CH_3_COOH.

### Comparison to other pretreatments under the same conditions

**Fig. 3:**
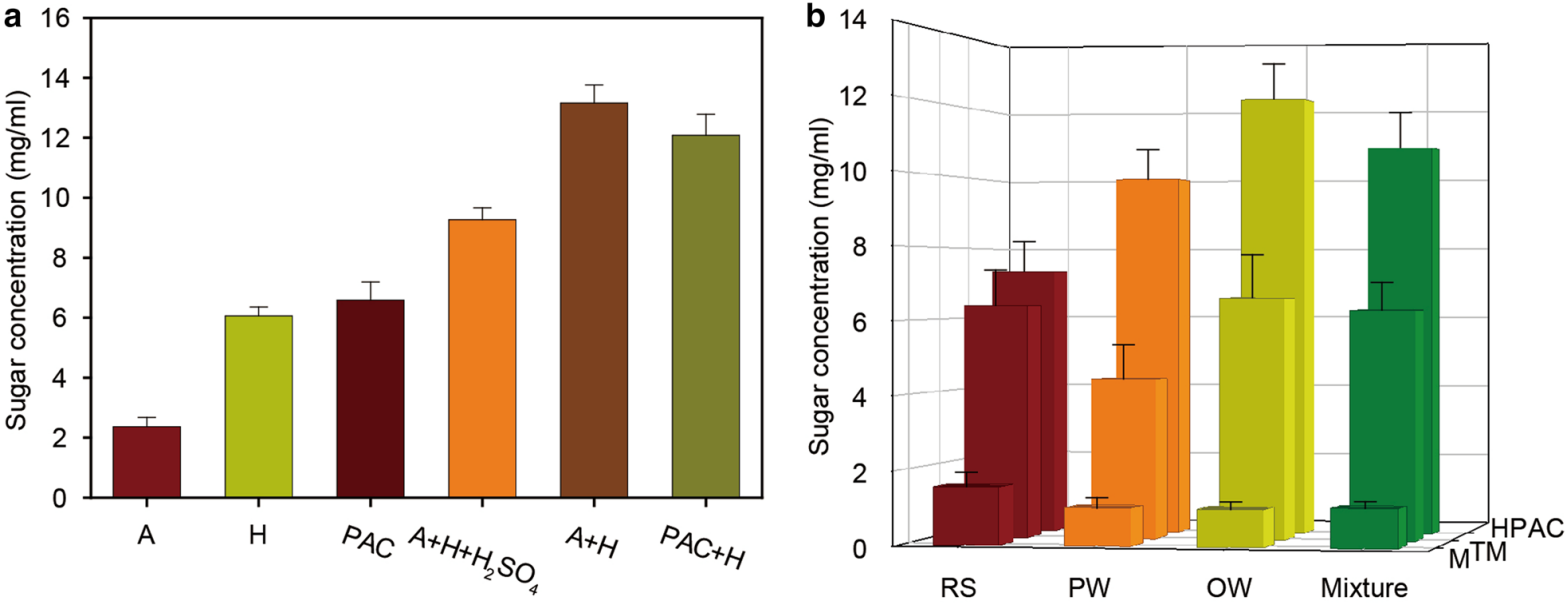
**a** Effects of different organo-solvent pretreatments on enzymatic hydrolysis of pine wood. (*A* acetic acid, *H* hydrogen peroxide, *PAC* peracetic acid). **b** Influence of different pretreatment methods on sugar yields (*RS* rice straw, *PW* pine wood, *OW* oak wood)

Previous studies on organo-solvent pretreatments have shown that the enzymatic digestibility of hydrogen peroxide [29, 30], acetic acid [31], peracetic acid (PAA, CH_3_CO_3_H) [17], and a H_2_O_2_—CH_3_COOH mixture effectively increases in the presence of catalysts such as sulfuric acid (H_2_SO_4_) [32, 33], titanium dioxide (TiO_2_) [34, 35], and sodium molybdate (Na_2_MoO_4_) [36]. Therefore, we tested different solvent conditions, such as those with CH_3_COOH, H_2_O_2_, CH_3_CO_3_H, and a CH_3_CO_3_H/H_2_O_2_ mixture. Compared to organo-solvent pretreatment under the same conditions, the HPAC pretreatment was more effective at increasing enzymatic digestibility (Fig. 3a). In addition, compared to a mechanical pretreatment (milling) and pretreatment with another acid (H_2_SO_4_), the HPAC pretreatment was more effective at converting all three biomass materials into glucose (Fig. 3b). Moreover, when a mixture of three lignocellulosic materials was treated, the HPAC pretreatment was very effective at increasing enzymatic digestibility (Fig. 3b). These results indicate that HPAC pretreatment is a highly efficient and effective process for converting lignocellulosic materials into fermentable sugars.

### Changes in chemical composition during pretreatment

A key question that we investigated was why the HPAC pretreatment is more effective than other processes. We reasoned that the main effect of HPAC pretreatment may be complete or nearly complete removal of lignin from the lignocellulosic biomass, leading to greatly improved enzymatic efficiency. The lignin in lignocellulosic cell walls restricts access of cellulase to cellulose, blocks progression of cleavage of cellulose chains by cellulases, and binds non-productively with cellulases, resulting in wasted enzymes. Lignin content and composition were analyzed to understand the impact of the HPAC pretreatment on delignification.

The compositions of the main chemical constituents in the three different biomass materials are provided in Additional file 3: Figure S3 and highlight the compositional differences in the feedstocks before and after HPAC pretreatment. All compositions were calculated based on the dry sample weight. The most significant compositional change was in lignin. The HPAC process removed 98.08 % of the acid-insoluble lignin from the pine wood feedstock. Lignin content decreased by 97.61 and 85.12 % in oak wood and rice straw, respectively. A relative increase in cellulose content was observed, compared to the initial raw material, due to the removal of other constituents such as lignin and ash. The low feedstock quality of rice straw was primarily due to its high ash and silica content compared to wood.

The yields of different reducing sugars, with respect to the increased pretreatment time for pine wood, are shown in Additional file 4: Figure S4. Glucose content was maximum (93.4 %) after 2 h of pretreatment. Lignin content dropped markedly by 98 % with an increase in pretreatment time to a maximum of 2 h (Additional file 5: Figure S5). Thus, the increased conversion of cellulose would lead to an increase in available sugar content in the hydrolyzate.

### Solid-state CP/MAS ^13^C NMR analysis

**Fig. 4:**
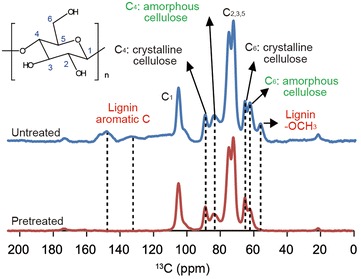
Solid ^13^C-NMR spectra for pretreated and untreated pine wood

Solid-state magic angle spinning carbon-13 nuclear magnetic resonance (CP/MAS ^13^C NMR) experiments were carried out on pine wood either pretreated with HPAC or not pretreated, to collect data on chemical composition. The spectra are shown in Fig. 4 and Additional file 6: Figure S6. The NMR resonances were assigned according to data from the literature [37–39]. The peaks at δ 61.9 and δ 64.8 ppm were assigned to the C-6 glucopyranosyl repeating units in cellulose. The cluster of resonances around the peaks at δ 72.2 and δ 75.8 ppm were assigned to C-2, C-3, and C-5. The peaks at δ 84.4 and δ 89.0 ppm were attributed to C-4, and the absorption peak at δ 105.0 ppm was assigned to C-1 of glucose in cellulose. The solid-state NMR of pretreated pine wood showed a decrease in signal intensities at both the C-6 and C-4 peaks of amorphous cellulose, indicating preferential degradation of the amorphous regions during the pretreatment. In particular, signals at δ 56 ppm and δ 130–155 ppm were associated with the methoxyl and aromatic groups of lignin, respectively, demonstrating that the HPAC pretreatment removed all lignin components.

### Fourier transform-infrared (FT-IR) analysis

FT-IR spectroscopy is frequently used to investigate the structure of constituents and the chemical changes in lignocellulosic biomass during pretreatment. Three representative chemical changes related to lignin removal based on the FT-IR analysis are shown in Additional file 7: Figure S7. The pretreated samples displayed significant decreases in band intensities at 1450 (C‒H deformation), 1508 (aromatic skeletal vibrations), and 1640 cm^−1^ (conjugated C=O stretch). This indicates that the HPAC pretreatment causes a breakdown of the aromatic structure of lignin.

### Lignin staining and brightness

Microscopic observations also provided evidence of changes in lignocellulosic materials during the HPAC treatment. To confirm whether lignin was removed, we observed lignin by counterstaining with phloroglucinol, a reagent widely used to contrast lignified cell walls. Lignified cell walls acquire a bright red color with this stain. Sections from HPAC-treated material did not stain with phloroglucinol-HCl, indicating the absence of lignin from cell walls (Additional file 8: Figure S8). This indicates that lignin content continues to decrease during HPAC treatment. Moreover, delignification of pine wood was further visually evaluated using a brightness analysis. Brightness improved by as much as 70.9 % after HPAC pretreatment (Additional file 9: Figure S9), which was attributable to removing lignin. Lignin is the main source of color in pulp, as a variety of chromophores are naturally present in wood.

### Morphological changes in biomass from HPAC pretreatment

The physical structure of biomass changes naturally after pretreatment, which can further affect enzymatic hydrolysis. Scanning electron microscopic (SEM) micrographs of pretreated and untreated pine wood were taken to study the changes in surface characteristics of the biomass. The SEM images clearly showed that untreated pine wood had a smooth and continuous surface. However, pores were present in the pretreated wood, which may have been due to high levels of removed residual lignin (Additional file 10: Figure S10). Moreover, cellulose fibrils appeared to have separated, suggesting enhanced enzymatic accessibility to cellulose. Similar results were obtained for oak wood and rice straw. These results indicate that HPAC pretreatment induces severe morphological changes in the plant cell walls studied and are in general agreement with significant lignin removal observed.

### Simultaneous saccharification and fermentation (SSF)

**Fig. 5:**
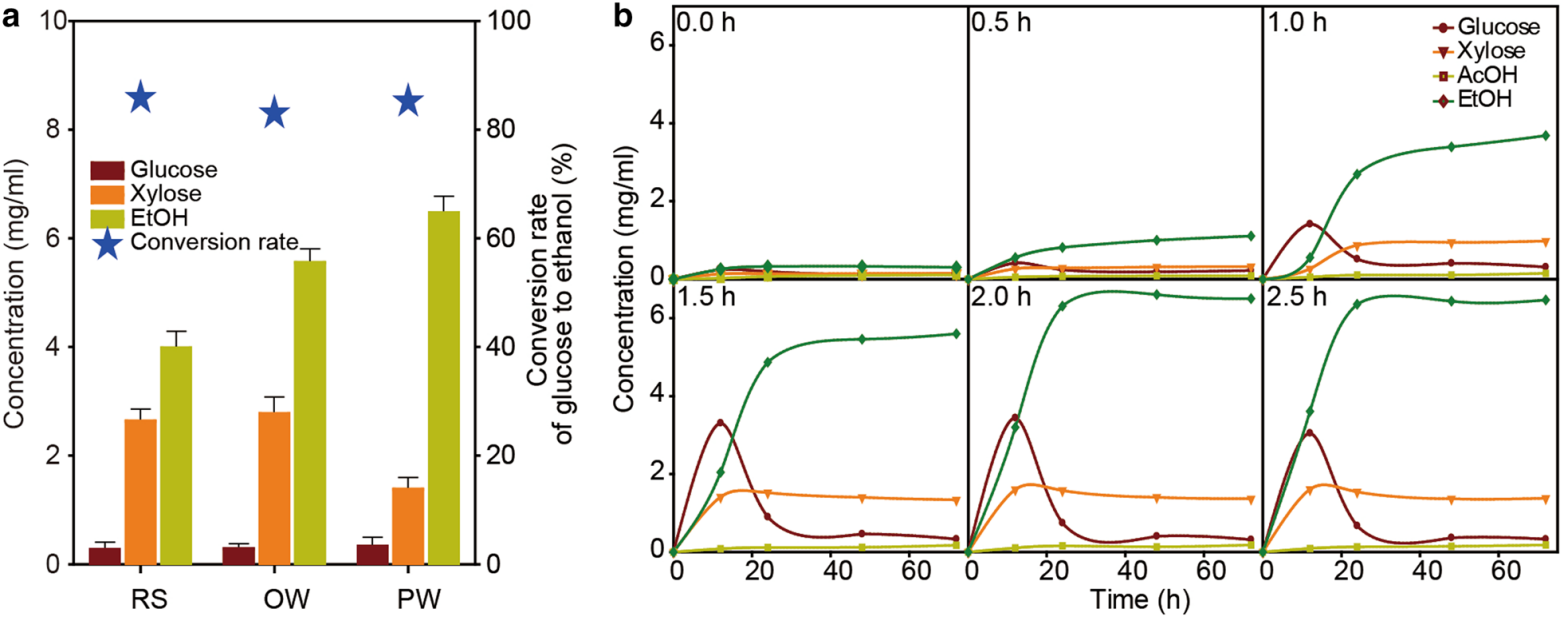
**a** Conversion rate of glucose to ethanol and the concentrations of sugars and ethanol. **b** Time course of sugar utilization and ethanol production by *Saccharomyces cerevisiae* from hydrolyzate using an enzyme mixture containing cellulase (10 FPU/g DM) and xylanase (20 IU/g DM) after the HPAC pretreatment. Note: *a* 0 h, *b* 0.5 h, *c* 1.0 h, *d* 1.5 h, *e* 2.0 h, and *f* 2.5 h of HPAC pretreatment time

The SSF process is one of the most promising processes for the production of ethanol from lignocellulosic biomass. The SSF process is carried out at faster rates, higher yields, and greater ethanol concentrations than is possible for the separate hydrolysis and fermentation (SHF) [40]. Thus, the productivity of ethanol was investigated by SSF of the three pretreated biomass materials to evaluate the efficiency of the HPAC pretreatment. Saccharification was achieved using crude cellulase and fermentation for 24 h by *Saccharomyces cerevisiae*, which were cultured at very near their optimum temperature (37 °C) with 10 FPU cellulase g^−1^ biomass and 20 IU xylanase g^−1^ biomass (Fig. 5a). The ethanol concentration in HPAC-pretreated pine wood reached 0.325 g/g biomass, based on enzymatic hydrolysis, assuming 87 % fermentation yield within a 24-h period (1.32 g ethanol/3.50 g glucose). Ethanol yields from pine wood and rice straw were 84 and 86 % of the theoretically expected values, respectively.

Glucose and xylose production and the conversion to ethanol depending on pretreatment and SSF time are shown in Fig. 5b and Additional files 11, 12: Figures S11, S12. Most of the glucose and xylose were released within 24 h. Glucose was converted into ethanol as quickly as it was produced, and most of the glucose produced was converted into ethanol within 24 h. However, xylose-to-ethanol conversion did not occur, because *S. cerevisiae* cannot ferment xylose into ethanol.

### Light micrographs after enzymatic hydrolysis

The effects of enzymatic hydrolysis on pine wood were further visually evaluated using light micrographs. After 24 h of enzymatic hydrolysis, pine cell walls were not observed in the cell wall structure (Additional file 13: Figure S13). These results could be explained, as least in part, by the extensive lignin removal during pretreatment, which increased enzyme accessibility to cellulose. In addition, the higher ethanol yield from the pretreated material was due to the increased quantities of reducing sugars in the fermenting medium.

### Inhibitor analysis

One of the disadvantages of acid pretreatment is the formation of inhibitory compounds. The mild conditions of the HPAC pretreatment reduced degradation of monosaccharides into furfural and 5-hydroxymethylfurfural (HMF), which are very toxic to fermenting organisms. We confirmed that no inhibitors existed in pretreated solid residues using high-performance liquid chromatography (Additional file 14: Figure S14). Effective removal of inhibitory compounds would allow the use of more severe pretreatment conditions, which can improve sugar yields and lead to more efficient fermentation.

### Overall mass balance

**Fig. 6:**
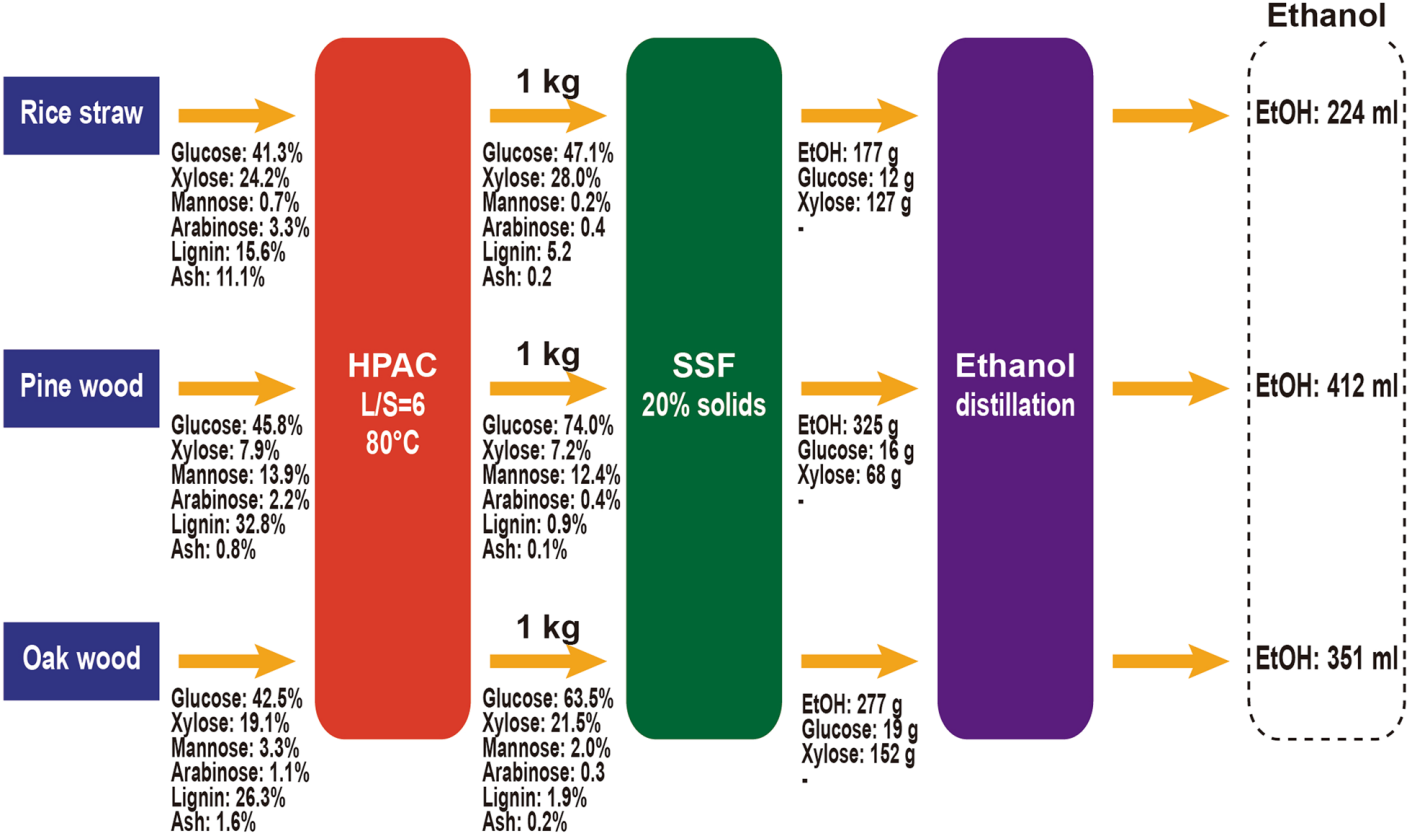
Material balances for 1 kg of HPAC-pretreated lignocellulosic biomass materials extrapolated from the results of 10.0-g-dry weight scale experiments

Using compositional analyses after each step, we created an overall mass balance for our operation, including the HPAC pretreatment and SSF steps (Fig. 6). The experiment used 5.25 kg of CH_3_COOH and 5.65 kg of H_2_O_2_ g^−1^ for pine wood (1:6 ratio of pine wood to solution at 80 °C for 2 h) for the pretreatment. After HPAC treatment, the composition of the recovered solid was 74.0 % cellulose, 20.0 % hemicelluloses, and 0.9 % lignin. The total amount of hemicelluloses expressed as sum of xylose, mannose, and arabinose. Notably, 97.2 % of the lignin was removed with HPAC pretreatment. After pretreatment, a substrate loading of 20 % (w/v) was chosen due to the well-known limiting effect of elevated substrate loading for SSF. Fermentation of the hydrolyzates by *S. cerevisiae* resulted in 412 mL ethanol kg^−1^ of biomass after 24 h, which was equivalent to 85.0 % of the maximum theoretical yield (based on the amount of glucose in the raw material). These results suggest that the cost of ethanol production from lignocellulosic biomass can be significantly reduced, as high sugar yields can be obtained with low enzyme loading.

## Conclusion

In summary, lignocellulosic cell walls are analogous to reinforced concrete, in which the steel rebar resembles cellulose and the concrete resembles the lignin matrix. Removing concrete from a reinforced concrete structure readily exposes and weakens the steel rebar. Similarly, delignification exposes the cellulose fibrils in cell walls, resulting in efficient enzymatic hydrolysis of cellulose. Highly effective removal of lignin by the HPAC pretreatment resulted in enhanced enzymatic accessibility of the substrate, leading to more efficient cellulose hydrolysis. Furthermore, the HPAC pretreatment led to less formation of fermentative inhibitory compounds. In addition, the HPAC pretreatment enables year-round operations to maximize the value of biomass feedstock supply chains. HPAC pretreatment of the three different types of lignocellulosic biomass materials (i.e., an herbaceous plant, a softwood, and a hardwood) greatly improved downstream saccharification and fermentation, which are important for obtaining a high yield of bioethanol. In conclusion, our new HPAC pretreatment method will contribute to more effective bioethanol production from a wide variety of lignocellulosic biomass materials, with the added benefit of year-round application.

## Methods

### Materials

Hydrogen peroxide, acetic acid, and sulfuric acid were purchased from Duksan Chemicals Co. (Seoul, Korea). Bio-Rad reagent for protein assay was obtained from Bio-Rad Laboratories (Hercules, CA, USA). The water used throughout this study was de-ionized and filtered using a US. Filter purification system. Oak wood and pine wood were obtained from a field in Chonnam National University, South Korea. Rice straw was obtained from a field in Mooan, South Korea, after being harvested for air dried at ambient temperature to equilibrium moisture content. The dried biomass were chopped into small pieces of ~2 cm in length with a cutter, ground with a wet-disk mill (particle size: 0.7  ±  0.2 cm) and stored for pretreatment.

### HPAC (hydrogen peroxide–acetic acid) pretreatment

The solution was prepared by mixing hydrogen peroxide and acetic acid (1:1; v/v). Ten grams each of the cellulosic wastes was treated separately with 100 mL of the reagent and kept at 80 °C for 2 h. After pretreatment, the reaction was carried out in a thermostatic water bath. The residues were collected and washed extensively with distilled water until neutral pH was reached, filtered, and dried at room temperature for 2 days.

- Ratios of hydrogen peroxide/acetic acid: 1:9, 2:8, 3:7, 4:6, 5:5, 6:4, 7:3, 8:2, and 9:1 (v/v)
- Temperature: 50, 60, 70, 80 and 90 °C
- Reaction time: 30, 60, 90, 120, and 150 min. Reaction time was started after sample s reached the desired temperature.

### Chemical composition analysis

After pretreatment, the biomass slurries were filtrated through Kimax™ Gooch 30-mL filtering crucibles, and the solid residue (WIS: water-insoluble solids) and WSF (water-soluble fraction) were separated. The WIS was subjected to enzymatic hydrolysis and the composition of WIS was analyzed. The chemical composition of holocellulose, lignin, organic solvent extractives and ash was analyzed using TAPPI Standard Methods [41]. Analyses of structural sugars (glucose, xylose, arabinose, mannose, galactose, and rhamnose) were conducted using a gas chromatograph [42].

### Histochemical analysis of lignin

Lignin was stained with Wiesner reagents where phloroglucinol in acidic conditions gives a red-pink product with mainly the cinnamaldehyde groups present in lignins. Fresh sections were left for 5 min in 2 % phloroglucinol in 95 % ethanol and mounted in 6 N HCl.

### Enzymatic hydrolysis of pretreated substrates

Enzymatic hydrolysis of WIS from pine wood, oak wood, and rice straw was carried out 2 % substrate consistency (2 g biomass per each 100 mL solution, 10 mM of sodium acetate buffer, pH 5.0) and 37 °C. The commercial enzymes used in this study were cellulase from *Trichoderma longibrachiatum* (C9748, Sigma-Aldrich) and xylanase from *Trichoderma longibrachiatum* (X2629, Sigma-Aldrich). Filter paper unit activity of cellulase was measured in terms of FPU/mL [43]. One filter paper unit (FPU) was defined as the amount of enzyme required to release 1 μmol of glucose from filter paper per min. Xylanase activity was measured on the basis of xylose released from birch wood xylan as a substrate and was expressed in terms of international units (IU)/mL. One IU was defined as the amount of enzyme required to release 1 μmol of xylose from birch wood xylan per min [44]. The activities of cellulase and xylanase were 79 kFP U/mL and 592 IU/mL, respectively. Hydrolysate samples were collected at different time intervals during 24 h. Reducing sugar were measured using a 3,5-dinitrosalicylic acid reagent and a standard glucose curve [45].

### Simultaneous saccharification and fermentation (SSF)

Simultaneous saccharification and fermentation were conducted for pretreated materials in a 5-mL total volume containing 2 % (w/v) dry matter, cellulase (10 FPU/g mass), xylanase (20 IU/g mass), 5 mg dry yeast (*S*. *cerevisiae* KCTC 7906), 0.1 % (w/v) yeast extract, 0.2 % (w/v) peptone, and 0.05 M citrate buffer (pH 4.8) at 37 °C for 24 h in a 15-mL conical tube. All experiments were performed in triplicate, and ethanol yield was calculated on the basis of total glucose content in the pretreated materials by dividing the quantity of ethanol produced by the total amount of glucose.

### Analysis of sugars and degradation compounds

During enzymatic hydrolysis and fermentation, sugars, ethanol, and degradation compound were monitored using HPLC equipped with a refractive index (RI) detector (Waters 2414, USA). A Rezex RPM column (300 × 7.8 mm; Phenomenex, Torrance, CA) was used for sugars (i.e., glucose, and xylose). HPLC-grade water was supplied at a flow rate of 0.6 mL/min as a mobile phase at a controlled temperature of 85 °C. Also, a Rezex ROA organic acid column (300  ×  7.8 mm; Phenomenex, Torrance, CA) was used for compound identification (i.e., ethanol, furfural, HMF, levulinic acid, and acetic acid). The temperatures of the column and detector were maintained at 65 and 40 °C, respectively, and 5 mM sulfuric acid was added to the mobile phase at a flow rate of 0.6 mL per min.

The total amount of lignin degradation compounds in WSF liquors was determined by the Folin-Ciocalteu method using phenol as a standard and reported as phenol equivalents (PE) [46, 47].

### Structural characterizations

The surface morphologies of the samples were examined using field-emission scanning electron microscopy (FE-SEM) with a JSM-7500 F (Jeol, Japan) instrument operating at a beam voltage of 3 kV. Prior to observation, each sample was dehydrated with a graded ethanol series and freeze-dried. The external surface of the sample was then sputter-coated with osmium suing a sputter-coater. The FT-IR spectrum of the lyophilized sample was performed with the ATR technique using a PerkinElmer Spectrum 400. The spectrum was scanned in a range from 4000 to 400 cm^−1^ by 4 scans with a resolution of 4 cm^−1^. Solid-state ^13^C-NMR spectroscopy utilizing CP-MAS was performed using a Bruker DMX-400 MHz NMR spectrometer.

## Additional files

10.1186/s13068-015-0419-4 Effects of different volume ratios of hydrogen peroxide/acetic acid. (*Conditions:* temperature, 80 °C; time, 2 h) A. *Substrate*: oak wood, B. *Substrate*: rice straw.
10.1186/s13068-015-0419-4 Effects of temperature on relative conversion to sugars (*Conditions*: volume ratio of hydrogen peroxide/acetic acid, 5:5; time, 2 h). A. *Substrate*: oak wood, B. *Substrate*: rice straw.
10.1186/s13068-015-0419-4 Chemical composition analysis of untreated and pretreated biomass (RS, untreated rice straw; Pre-RS, pretreated rice straw; OW, untreated oak wood; Pre-OW, pretreated oak wood; PW, untreated pine wood; and Pre-PW, pretreated pine wood). Values are percentages on a dry matter basis.
10.1186/s13068-015-0419-4 Effects of pretreatment time on the yields of different reducing sugars. A. *Substrate*: pine wood, B. *Substrate*: oak wood, C. *Substrate*: rice straw.
10.1186/s13068-015-0419-4 Effects of reaction time on lignin content.
10.1186/s13068-015-0419-4 Solid ^13^C-NMR spectra of pretreated and non-pretreated oak wood.
10.1186/s13068-015-0419-4 FT-IR spectra of (A) oak wood and (B) pine wood.
10.1186/s13068-015-0419-4 (A) Distribution of lignin in pretreated and untreated pine wood: (a) untreated pine wood, (b) untreated pine wood after phloroglucinol staining, (c) pretreated pine wood, (d) pretreated pine wood after phloroglucinol staining. (B) Distribution of lignin in pretreated and non-pretreated oak wood. (a) untreated oak wood, (b) untreated oak wood after phloroglucinol staining, (c) pretreated oak wood, (d) pretreated oak wood after phloroglucinol staining.
10.1186/s13068-015-0419-4 Effect of brightness on reaction time.
10.1186/s13068-015-0419-4 (A) Scanning electron micrographs of treated and untreated pine wood. (a) Untreated pine wood at low magnification; (b) untreated pine wood at high magnification; (c) pretreated pine wood at low magnification; (d) pretreated pine wood at high magnification. (B) Scanning electron micrographs of treated and unpretreated oak wood and rice straw. (a) untreated oak wood, (b) untreated rice straw, (c) treated oak wood, (d) treated rice straw.
10.1186/s13068-015-0419-4 Time course of sugar utilization and ethanol production by *Saccharomyces cerevisiae* from hydrolyzate using an enzyme mixture containing cellulase (10 FPU/g DM) and xylanase (20 IU/g DM) after the HPAC pretreatment. Note: (a) 0 h, (b) 0.5 h, (c) 1.0 h, (d) 1.5 h, (e) 2.0 h, and (f) 2.5 h of HPAC pretreatment time. *Substrate*: oak wood.
10.1186/s13068-015-0419-4 Time course of sugar utilization and ethanol production by *Saccharomyces cerevisiae* from hydrolyzate using an enzyme mixture containing cellulase (10 FPU/g DM) and xylanase (20 IU/g DM) after the HPAC pretreatment. Note: (a) 0 h, (b) 0.5 h, (c) 1.0 h, (d) 1.5 h, (e) 2.0 h, and (f) 2.5 h of HPAC pretreatment time. *Substrate*: rice straw.
10.1186/s13068-015-0419-4 Light micrographs of pretreated (A) pine wood and (B) oak wood. (a) before and (b) after saccharification.
10.1186/s13068-015-0419-4 HPLC spectra of the simultaneous saccharification and fermentation (SSF) extracts. AA, acetic acid; Ff, Furfural; G1, glucose; G2, cellobiose; HMF, 5-hydroxymethylfurfural; SA, succinic acid; X1, xylose; Xt, xylitol.

## Abbreviations

HPAC: hydrogen peroxide–acetic acid
PAA: peracetic acid
NMR: nuclear magnetic resonance
FT-IR: Fourier transform-infrared
FE-SEM: field-emission scanning electron microscopy
SSF: Simultaneous saccharification and fermentation
*S. cerevisiae*: *Saccharomyces cerevisiae*
HPLC: high-performance liquid chromatography
HMF: 5-hydroxymethylfurfural
WIS: water-insoluble solids
WSF: water-soluble fraction
FPU: filter paper unit
IU: international units
RI: refractive index
RS: rice straw
PW: pine wood
OW: oak wood
