# Supplementary figures and images for: Lignocellulose conversion for biofuel: a new pretreatment greatly improves downstream biocatalytic hydrolysis of various lignocellulosic materials
Source: Biotechnol Biofuels. 2015 Dec 24;8:228. doi: 10.1186/s13068-015-0419-4 (PMC4690250; doi:10.1186/s13068-015-0419-4)

## Additional file 1: Figure S1

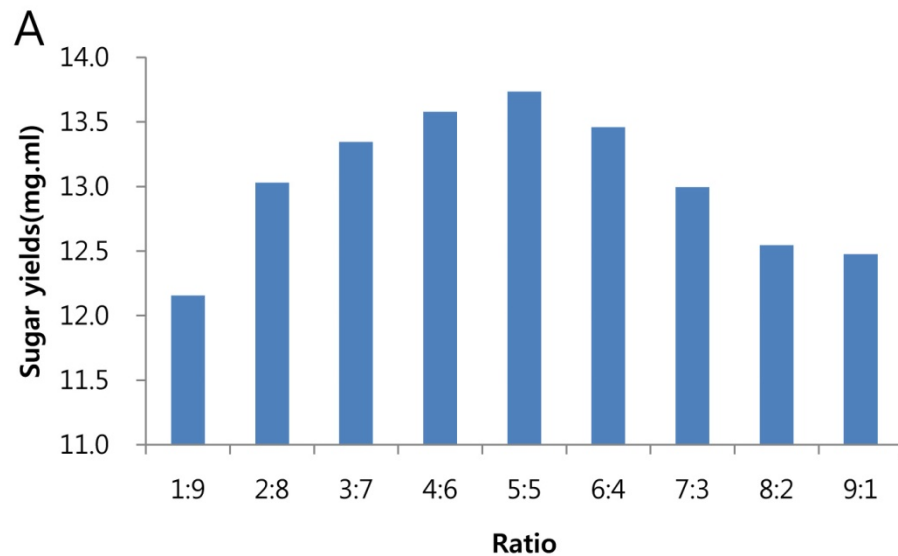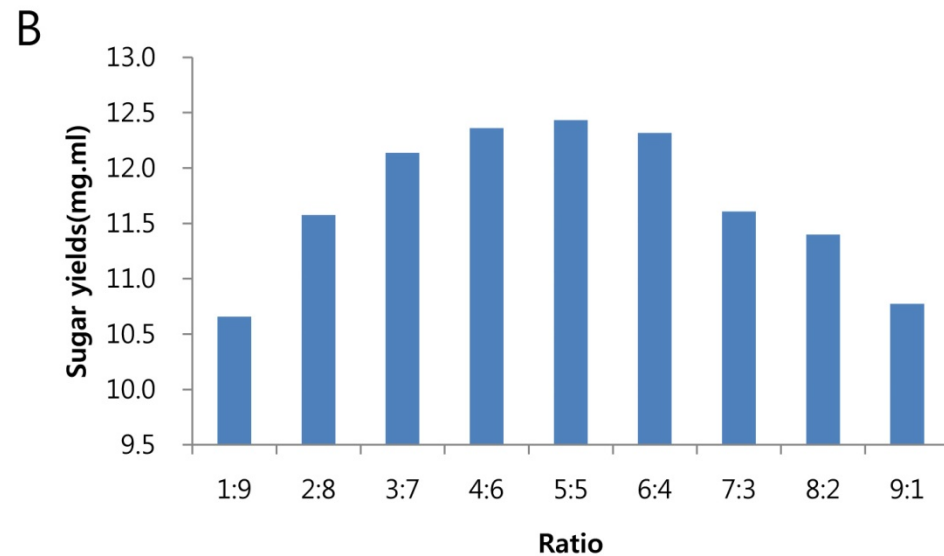

Supplement: Supplementary file 1 — 10.1186/s13068-015-0419-4 Effects of different volume ratios of hydrogen peroxide/acetic acid. (Conditions: temperature, 80 °C; time, 2 h) A. Substrate: oak wood, B. Substrate: rice straw. [file 13068_2015_419_MOESM1_ESM.pdf]

## Additional file 2: Figure S2

A

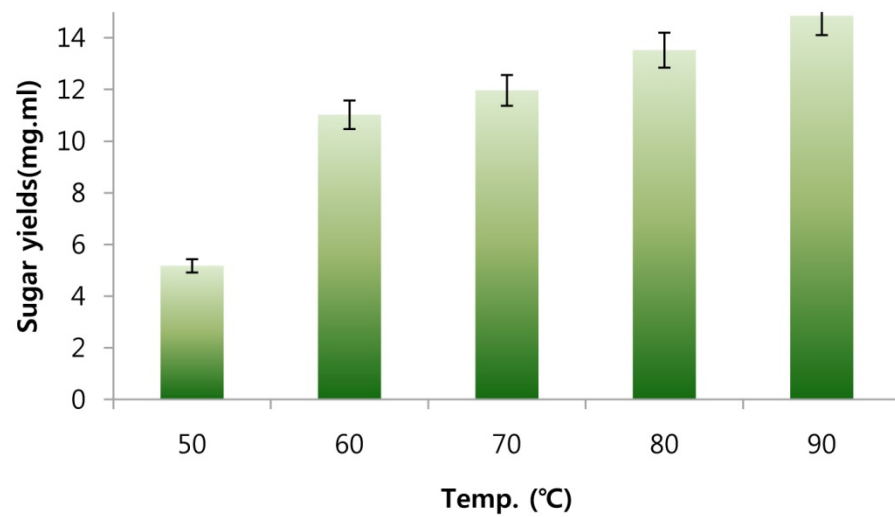

B

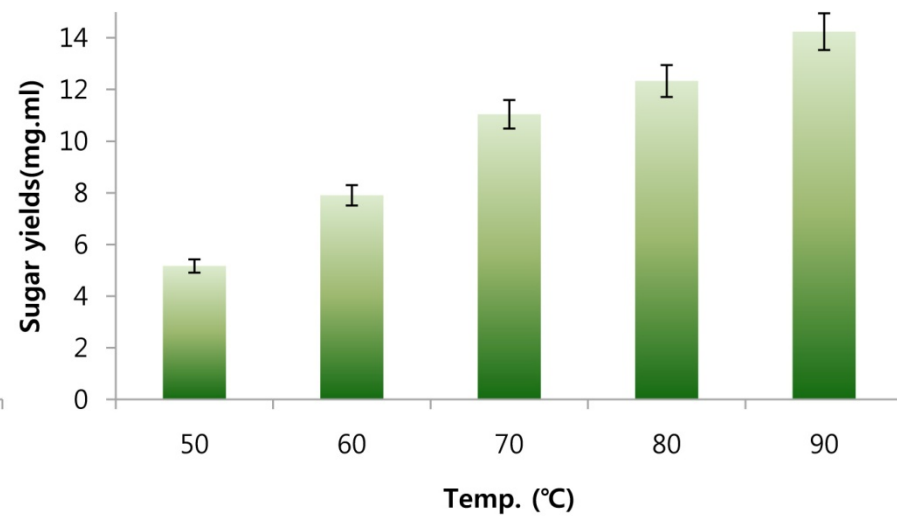

Supplement: Supplementary file 2 — 10.1186/s13068-015-0419-4 Effects of temperature on relative conversion to sugars (Conditions: volume ratio of hydrogen peroxide/acetic acid, 5:5; time, 2 h). A. Substrate: oak wood, B. Substrate: rice straw. [file 13068_2015_419_MOESM2_ESM.pdf]

Additional file 3: Figure S3

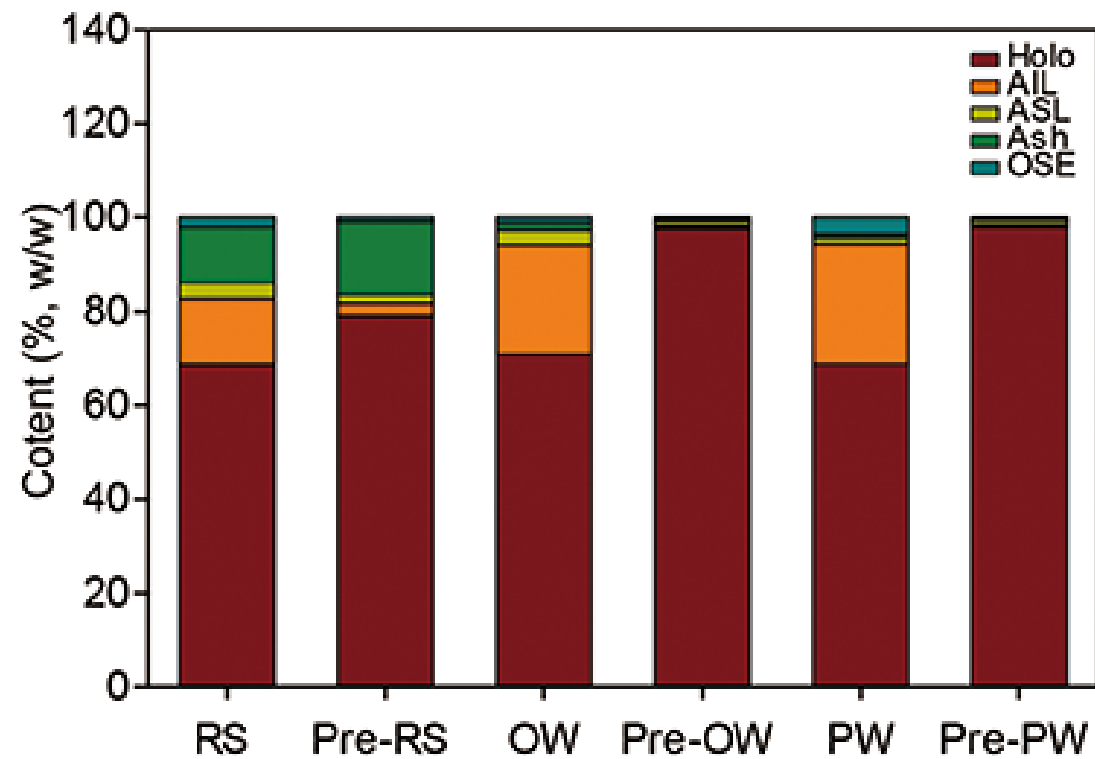

Supplement: Supplementary file 3 — 10.1186/s13068-015-0419-4 Chemical composition analysis of untreated and pretreated biomass (RS, untreated rice straw; Pre-RS, pretreated rice straw; OW, untreated oak wood; Pre-OW, pretreated oak wood; PW, untreated pine wood; and Pre-PW, pretreated pine wood). Values are percentages on a dry matter basis. [file 13068_2015_419_MOESM3_ESM.pdf]

## Additional file 4: Figure S4

**A**

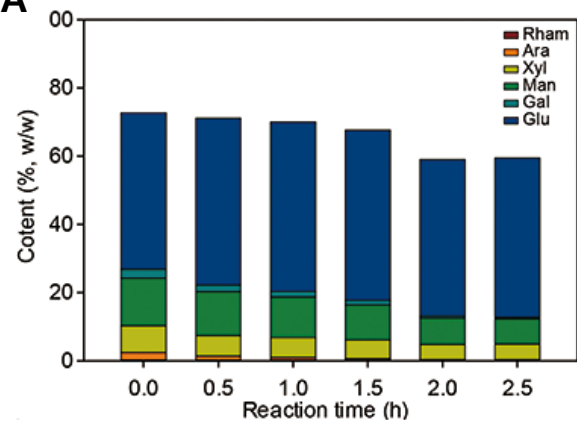

**B**

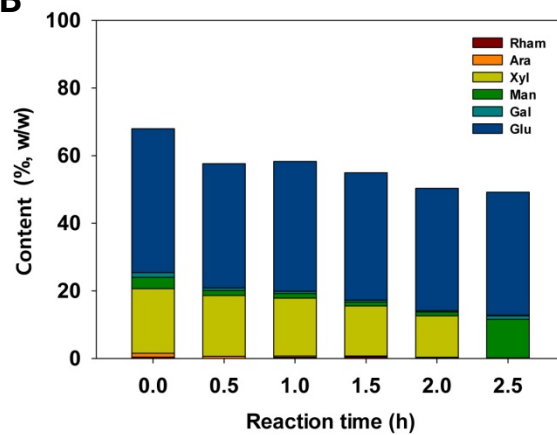

**C**

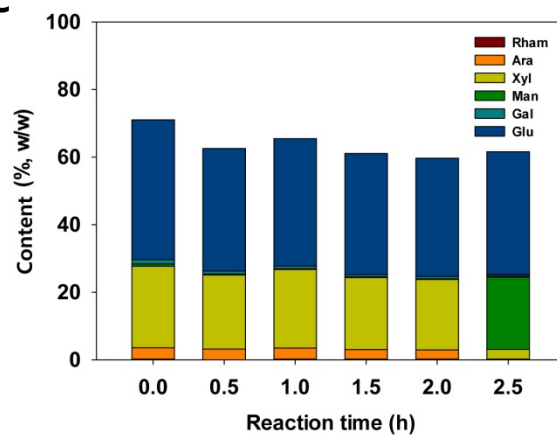

Supplement: Supplementary file 4 — 10.1186/s13068-015-0419-4 Effects of pretreatment time on the yields of different reducing sugars. A. Substrate: pine wood, B. Substrate: oak wood, C. Substrate: rice straw. [file 13068_2015_419_MOESM4_ESM.pdf]

Additional file 5: Figure S5

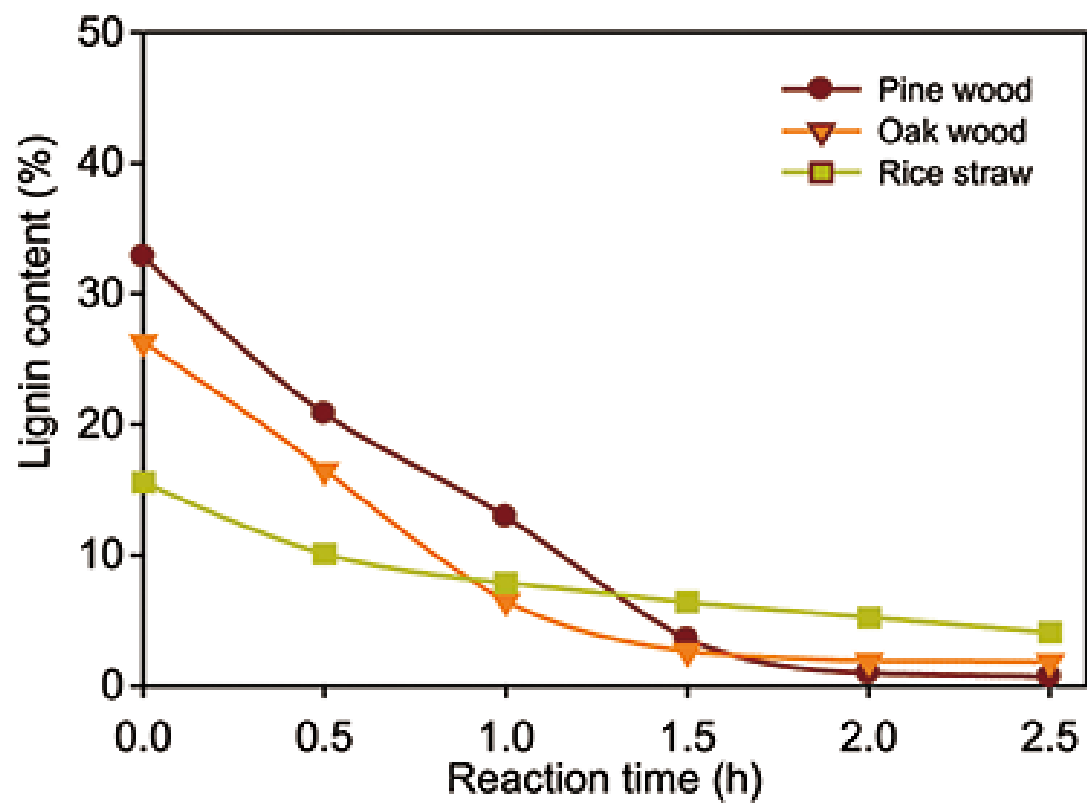

Supplement: Supplementary file 5 — 10.1186/s13068-015-0419-4 Effects of reaction time on lignin content. [file 13068_2015_419_MOESM5_ESM.pdf]

## Additional file 6: Figure S6

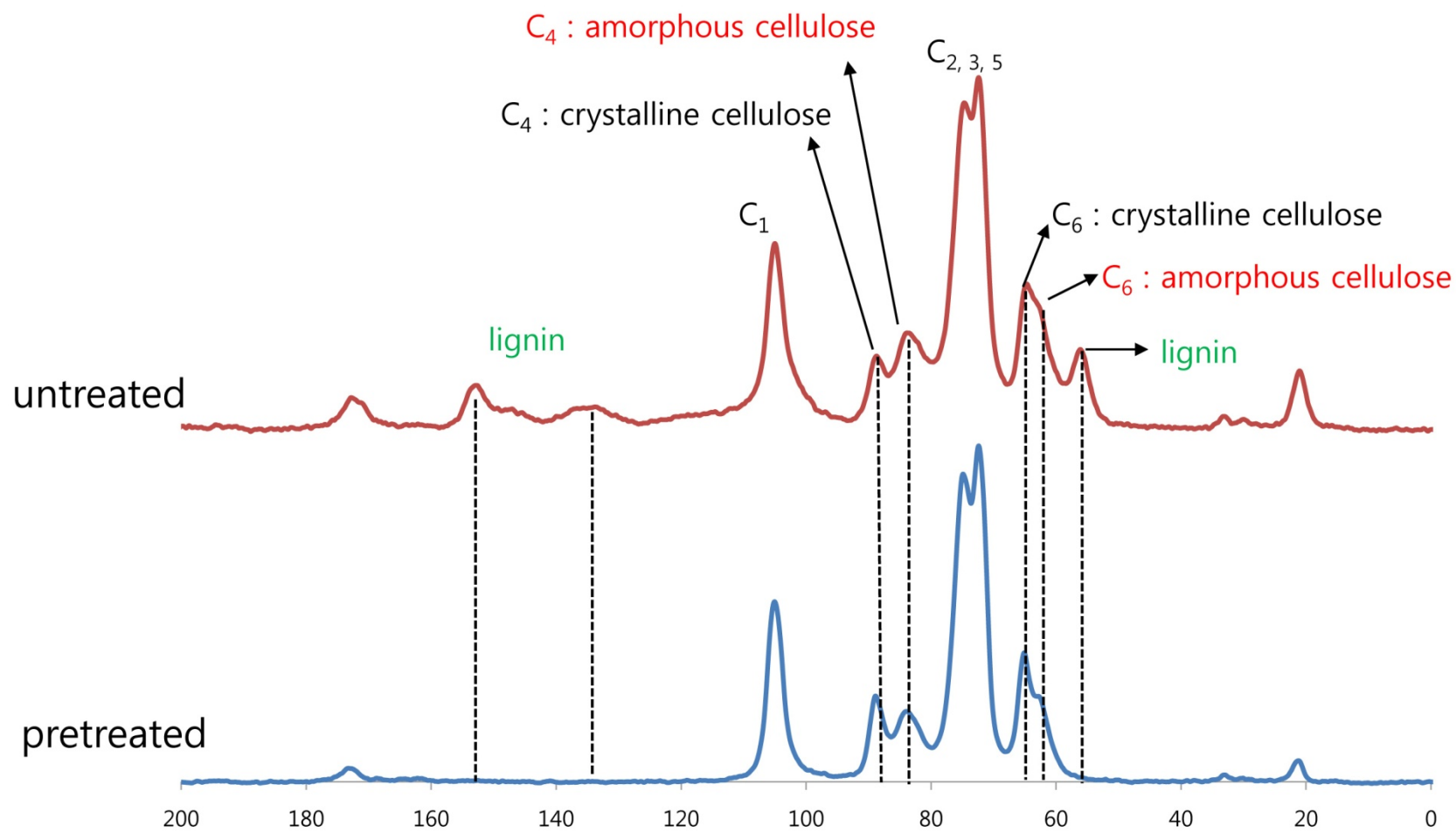

Supplement: Supplementary file 6 — 10.1186/s13068-015-0419-4 Solid 13C-NMR spectra of pretreated and non-pretreated oak wood. [file 13068_2015_419_MOESM6_ESM.pdf]

## Additional file 8: Figure S8

**A**

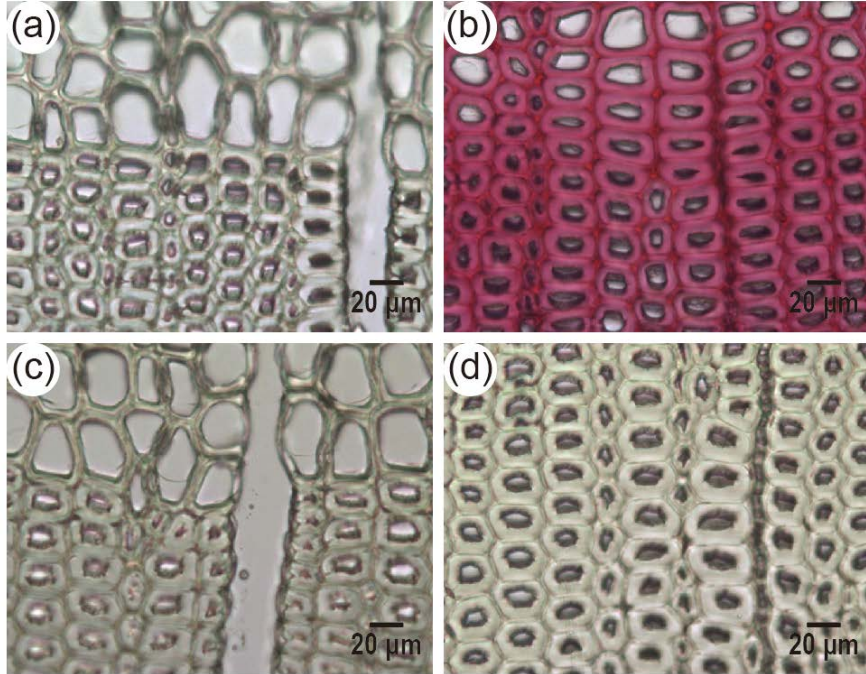

**B**

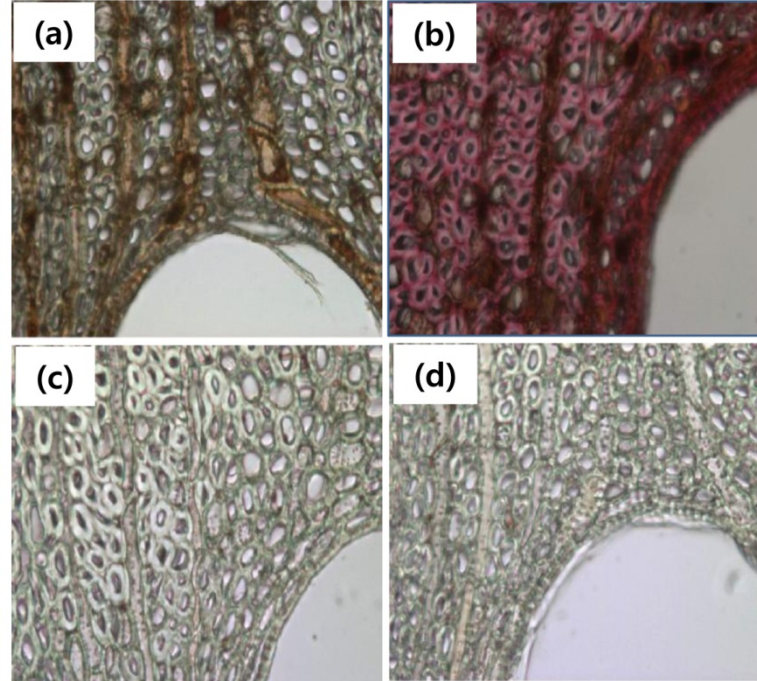

Supplement: Supplementary file 8 — 10.1186/s13068-015-0419-4 (A) Distribution of lignin in pretreated and untreated pine wood: (a) untreated pine wood, (b) untreated pine wood after phloroglucinol staining, (c) pretreated pine wood, (d) pretreated pine wood after phloroglucinol staining. (B) Distribution of lignin in pretreated and non-pretreated oak wood. (a) untreated oak wood, (b) untreated oak wood after phloroglucinol staining, (c) pretreated oak wood, (d) pretreated oak wood after phloroglucinol staining. [file 13068_2015_419_MOESM8_ESM.pdf]

## Additional file 9: Figure S9

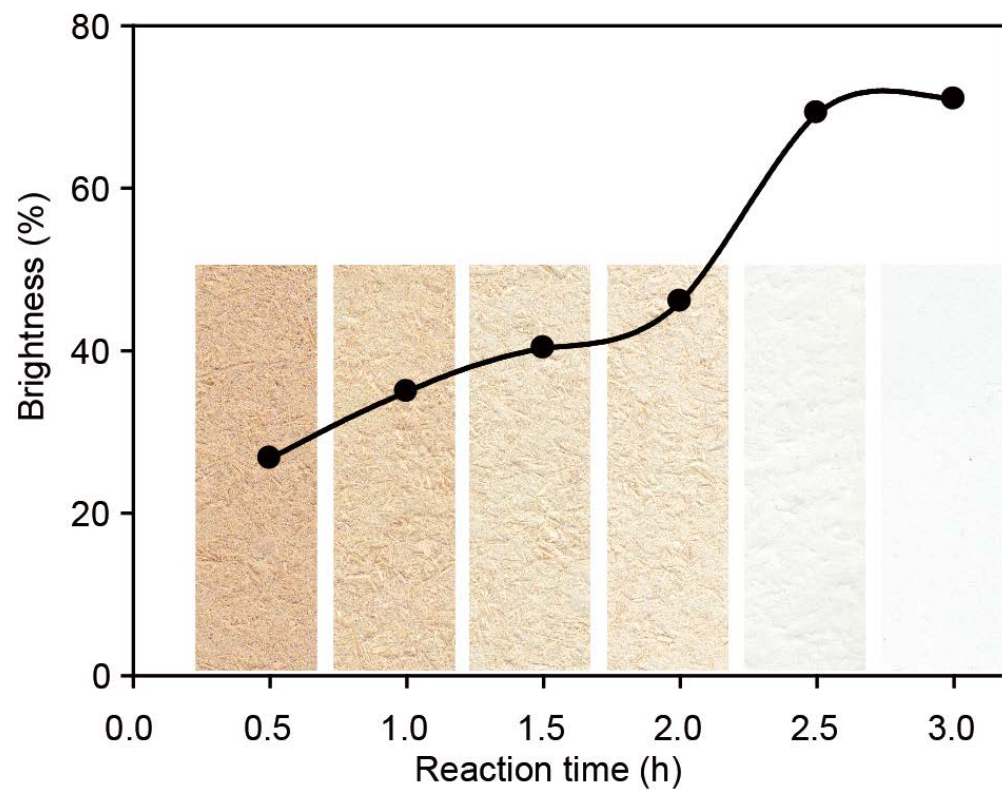

Supplement: Supplementary file 9 — 10.1186/s13068-015-0419-4 Effect of brightness on reaction time. [file 13068_2015_419_MOESM9_ESM.pdf]

# Additional file 10: Figure S10

**A**

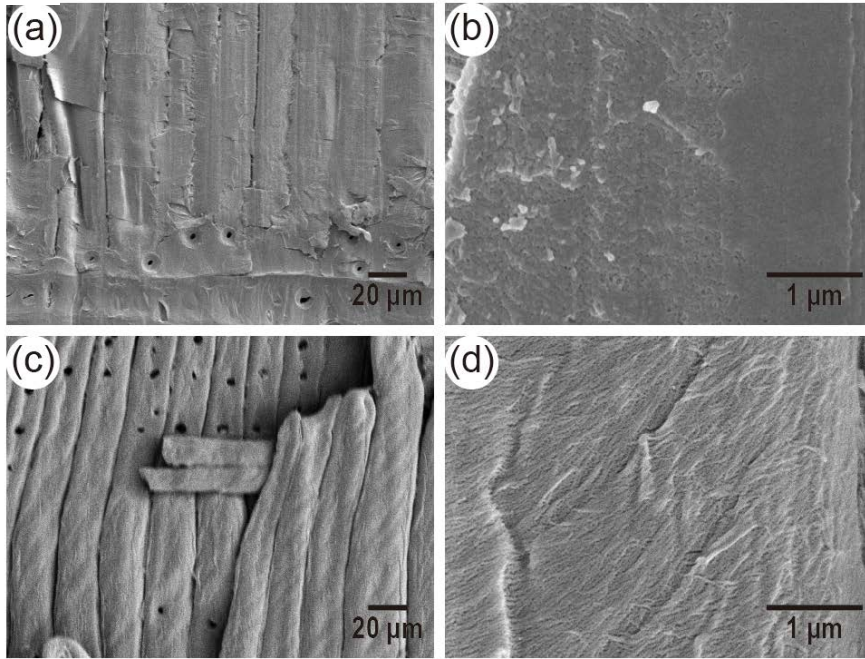

**B**

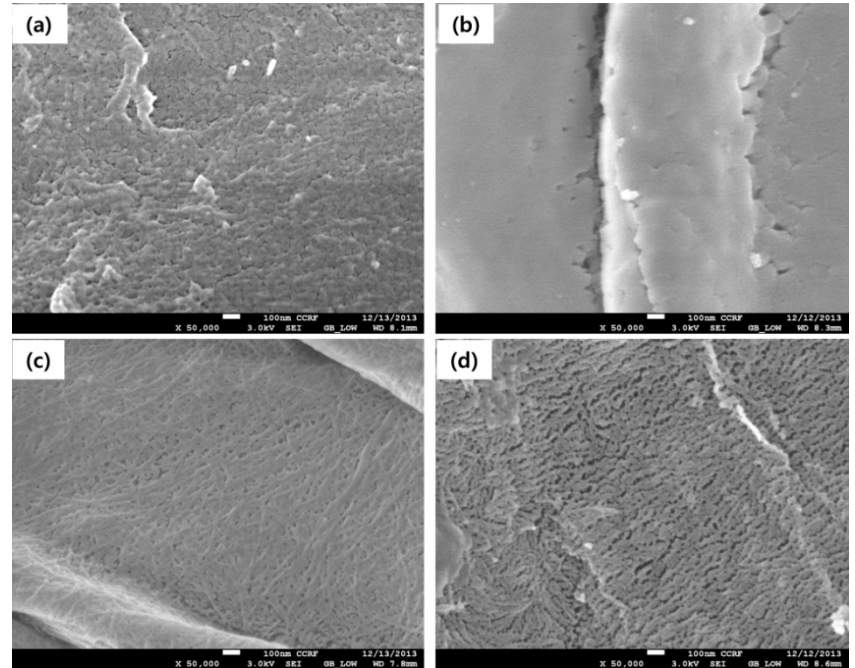

Supplement: Supplementary file 10 — 10.1186/s13068-015-0419-4 (A) Scanning electron micrographs of treated and untreated pine wood. (a) Untreated pine wood at low magnification; (b) untreated pine wood at high magnification; (c) pretreated pine wood at low magnification; (d) pretreated pine wood at high magnification. (B) Scanning electron micrographs of treated and unpretreated oak wood and rice straw. (a) untreated oak wood, (b) untreated rice straw, (c) treated oak wood, (d) treated rice straw. [file 13068_2015_419_MOESM10_ESM.pdf]

## Additional file 11: Figure S11

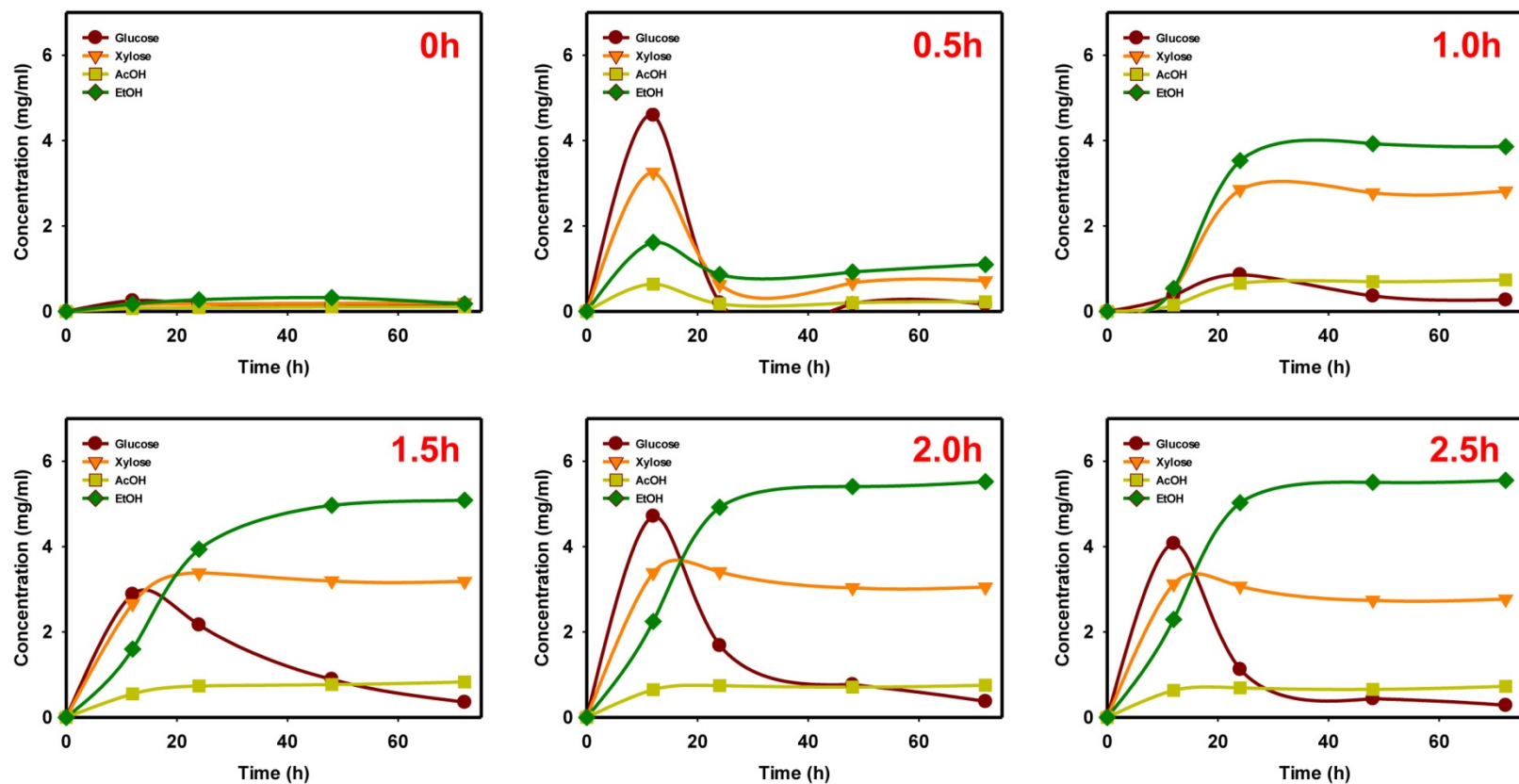

Supplement: Supplementary file 11 — 10.1186/s13068-015-0419-4 Time course of sugar utilization and ethanol production by Saccharomyces cerevisiae from hydrolyzate using an enzyme mixture containing cellulase (10 FPU/g DM) and xylanase (20 IU/g DM) after the HPAC pretreatment. Note: (a) 0 h, (b) 0.5 h, (c) 1.0 h, (d) 1.5 h, (e) 2.0 h, and (f) 2.5 h of HPAC pretreatment time. Substrate: oak wood. [file 13068_2015_419_MOESM11_ESM.pdf]

## Additional file 12: Figure S12

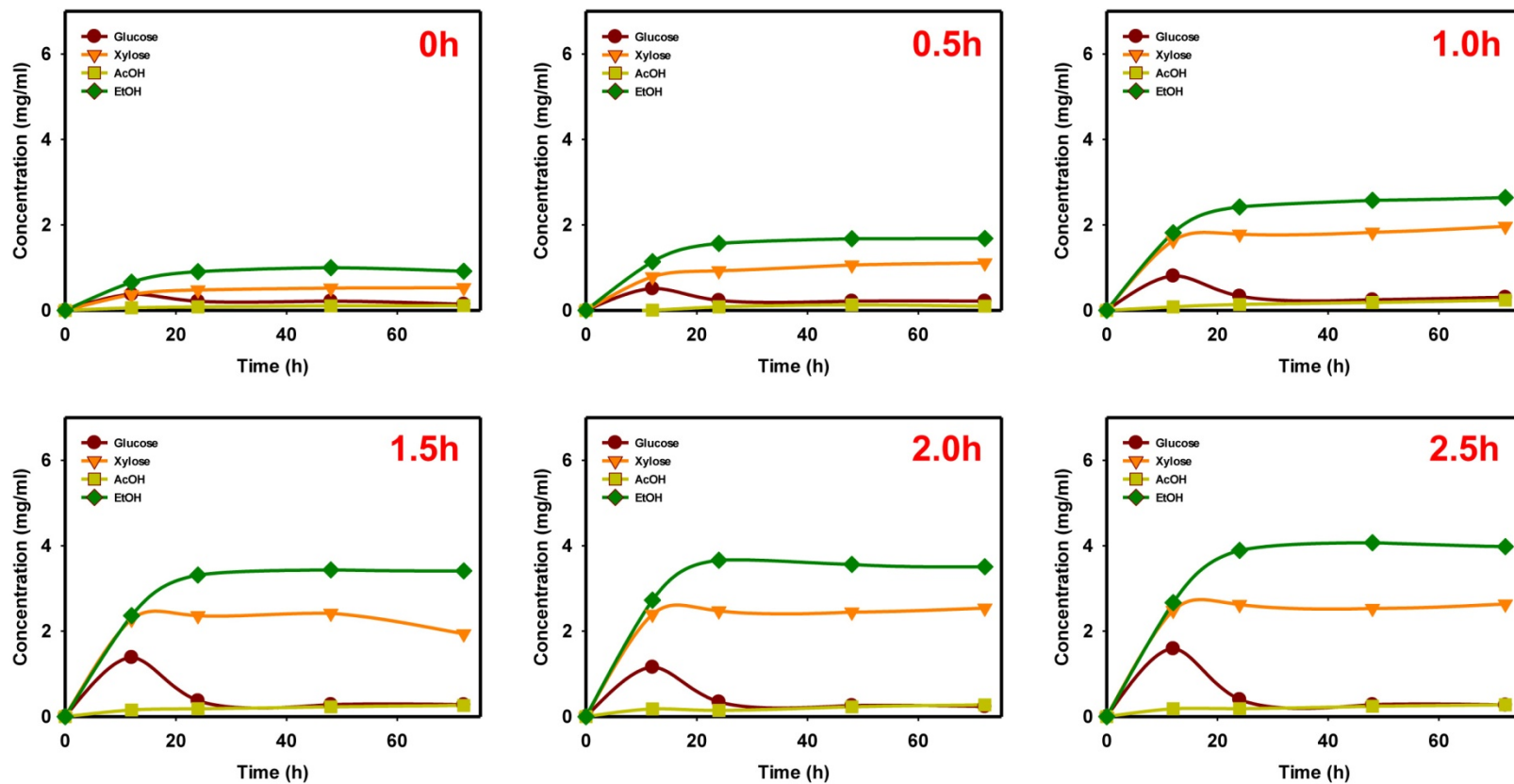

Supplement: Supplementary file 12 — 10.1186/s13068-015-0419-4 Time course of sugar utilization and ethanol production by Saccharomyces cerevisiae from hydrolyzate using an enzyme mixture containing cellulase (10 FPU/g DM) and xylanase (20 IU/g DM) after the HPAC pretreatment. Note: (a) 0 h, (b) 0.5 h, (c) 1.0 h, (d) 1.5 h, (e) 2.0 h, and (f) 2.5 h of HPAC pretreatment time. Substrate: rice straw. [file 13068_2015_419_MOESM12_ESM.pdf]

Additional file 13: Figure S13

**A**

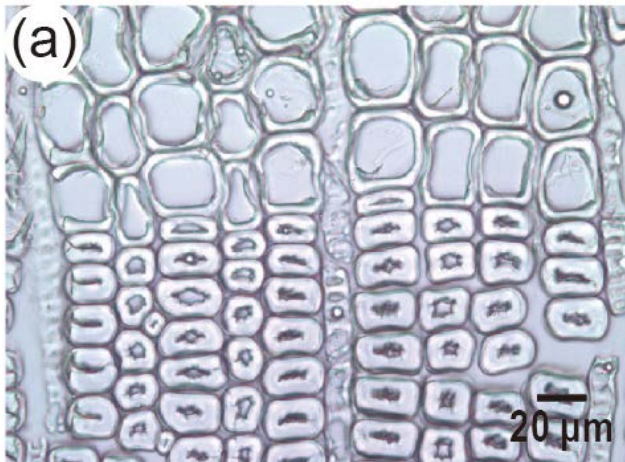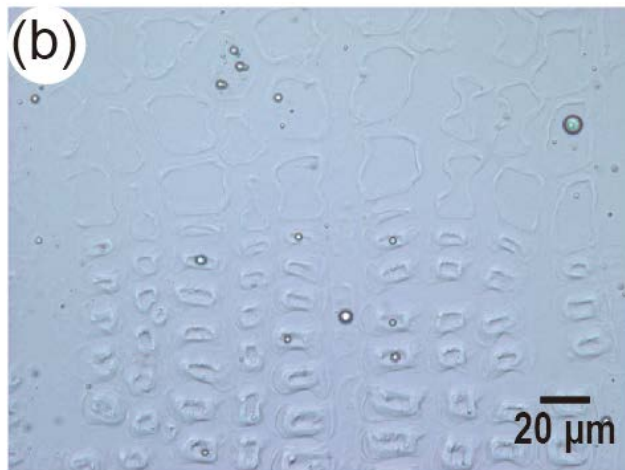

**B**

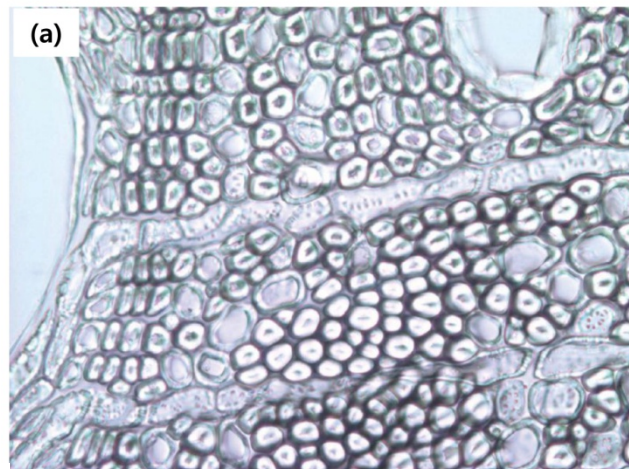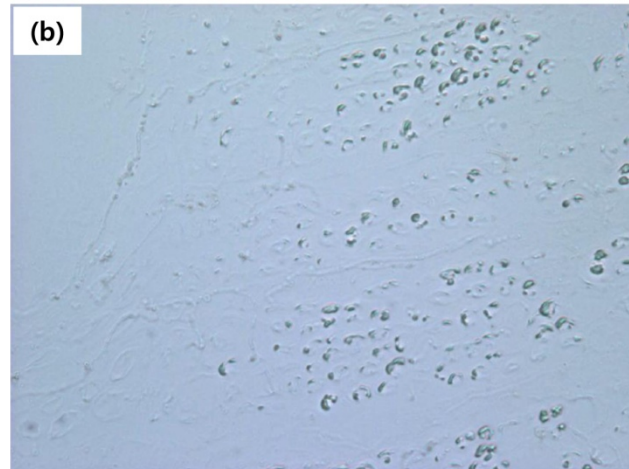

Supplement: Supplementary file 13 — 10.1186/s13068-015-0419-4 Light micrographs of pretreated (A) pine wood and (B) oak wood. (a) before and (b) after saccharification. [file 13068_2015_419_MOESM13_ESM.pdf]

## Additional file 14: Figure S14

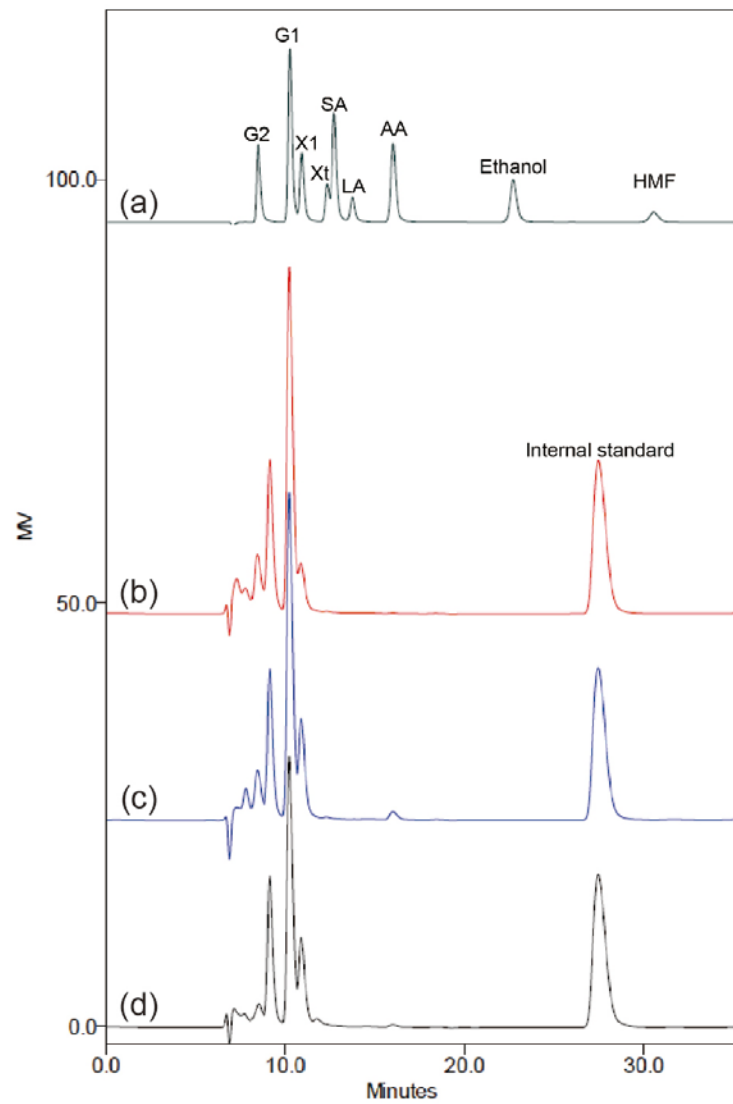

Supplement: Supplementary file 14 — 10.1186/s13068-015-0419-4 HPLC spectra of the simultaneous saccharification and fermentation (SSF) extracts. AA, acetic acid; Ff, Furfural; G1, glucose; G2, cellobiose; HMF, 5-hydroxymethylfurfural; SA, succinic acid; X1, xylose; Xt, xylitol. [file 13068_2015_419_MOESM14_ESM.pdf]
