# Supplementary material for: Lignocellulose conversion for biofuel: a new pretreatment greatly improves downstream biocatalytic hydrolysis of various lignocellulosic materials
Source: Biotechnol Biofuels. 2015 Dec 24;8:228. doi: 10.1186/s13068-015-0419-4 (PMC4690250; doi:10.1186/s13068-015-0419-4)
Supplement: Supplementary file 7 — 10.1186/s13068-015-0419-4 FT-IR spectra of (A) oak wood and (B) pine wood. [file 13068_2015_419_MOESM7_ESM.pdf]

Additional file 7: Figure S7

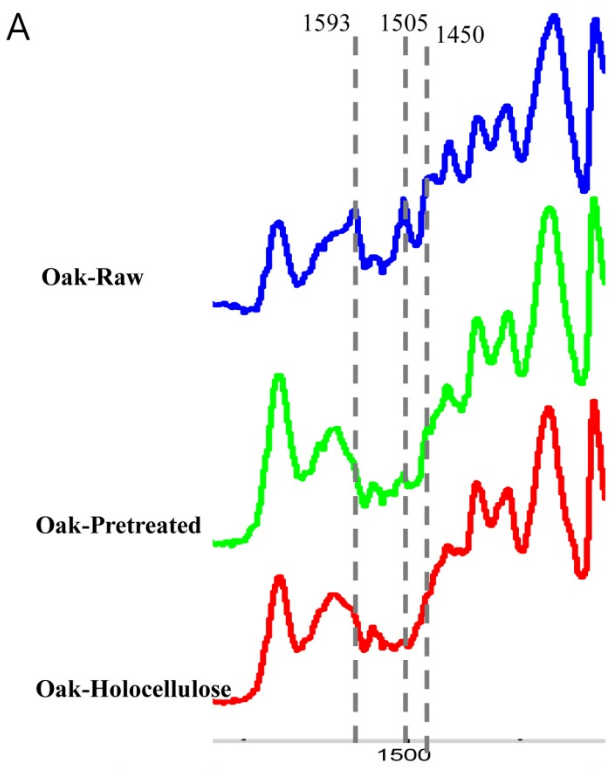

1450	C-H defoprmantion:<br>aromatic skeletal vibration of benzene ring in lignin
1505	aromatic skeletal vibration of benzene ring in lignin
1593	C=O stretching conjugated to the aromatic ring

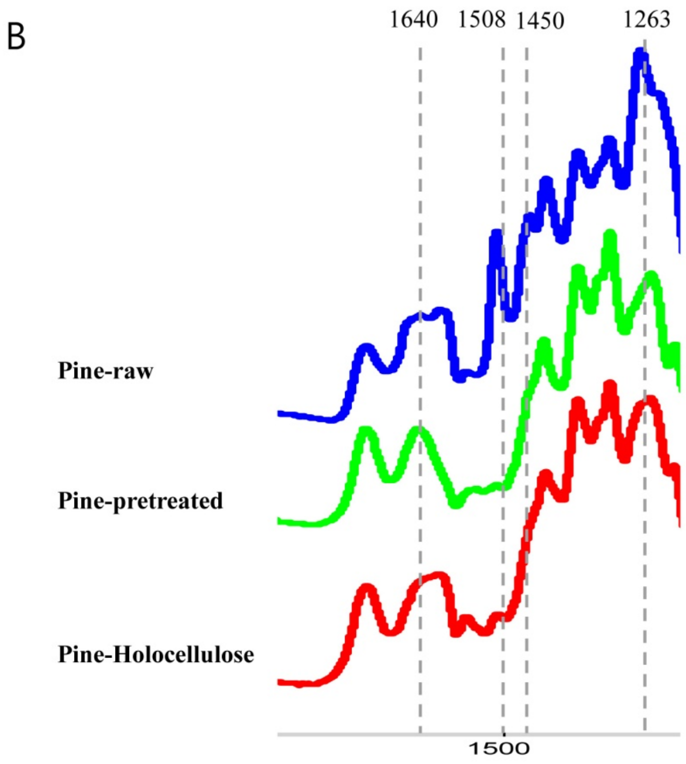

Band, cm-1	Assignment
1263	G ring breathing with carbonyl stretching
1450	C–H deformation in lignin
1508	aromatic skeletal vibrations in lignin
1640	conjugated C=O stretch, Oxidation of component (Zhao, J chem technol biotechenolog 83:950-956 (2008)
